# Multi-Center Analysis of Predictive Factors of Enteral Autonomy and Risk Factors of Complications of Pediatric Intestinal Failure in China

**DOI:** 10.3389/fped.2022.813865

**Published:** 2022-02-02

**Authors:** Weiwei Jiang, Guanglin Chen, Ying Wang, Wei Zhong, Chonggao Zhou, Jie Zhang, Xiaofeng Lv, Chunxia Du, Zhongxian Zhu, Qiming Geng, Weibing Tang

**Affiliations:** ^1^Department of Pediatric Surgery, Children's Hospital of Nanjing Medical University, Nanjing, China; ^2^Division of Pediatric Gastroenterology and Nutrition, Shanghai Jiaotong University School of Medicine Xinhua Hospital, Shanghai, China; ^3^Department of Neonatal Surgery, Guangzhou Women and Children's Medical Center, Guangzhou, China; ^4^Department of Neonatal Surgery, Hunan Children's Hospital, Changsha, China

**Keywords:** pediatric intestinal failure, enteral autonomy, intestinal failure-associated liver disease, catheter-related bloodstream infections, China

## Abstract

**Objectives:**

The aim of this study was to identify predictors for enteral autonomy and intestinal failure (IF)-related complications and evaluate the outcomes of a multi-center pediatric cohort in China.

**Methods:**

The medical records of pediatric patients with IF treated at four medical centers in China from January 1, 2012 to November 31, 2020 were retrospectively reviewed. Enteral autonomy was defined as sustained growth and cessation of parenteral nutrition for >90 days. Multivariate logistic regression analysis was used to identify factors predictive of enteral autonomy and the risk factors of complications, such as IF-associated liver disease (IFALD) and catheter-related bloodstream infection (CRBSI).

**Results:**

The study cohort of 92 pediatric patients with IF included 71 (77%) who underwent surgery and 21 (23%) who received non-surgical treatment. Eventually, 63 (68.5%) patients achieved enteral autonomy by the end of the follow-up period. Multivariate logistic regression analysis indicated that longer duration of parenteral nutrition (PN), sepsis, and non-breastfeeding were risk factors for enteral autonomy. When considering the detailed intraoperative data, the presence of an ileocecal valve (ICV) and greater residual small bowel (RSB) length were reaffirmed as predictors of achieving enteral autonomy. Medium/long-chain (MCT/LCT) lipids or sepsis were identified as negative predictors for IFALD. Univariate analysis revealed that the use of MCT/LCT lipids was associated with a greater likelihood of CRBSI.

**Conclusion:**

In this cohort, enteral autonomy was achieved at a percentage of 68.5%, and the risk factors for not achieving enteral autonomy were a longer duration of PN, sepsis, and non-breastfeeding. The presence of an ICV and a greater RSB length were important predictors of achieving enteral autonomy.

## Introduction

Intestinal failure (IF) refers to dysfunction of intestinal digestion and absorption, resulting in the inability of the intestine to fully absorb nutrients and liquids to meet the needs for growth and survival. The symptoms of IF include severe diarrhea, loss of water and electrolytes, acid/alkali balance disorder, malnutrition, and weight loss (1). The most common cause of IF in children is short bowel syndrome due to resection or congenital intestinal loss, intestinal motility disorders, and mucosal enteropathy (2–4).

Pediatric IF is a complex disease that was often fatal. However, the establishment of intestinal rehabilitation by multidisciplinary teams and improvements in nursing care and nutritional support have decreased the mortality rate over the past decade from 30% (5) to 10–15% (6–9). However, mortality associated with IF remains relatively high in many countries due to difficulties with treatment.

The main goals of intestinal rehabilitation are to promote intestinal adaptation and enteral autonomy, which is achieved by maintaining sufficient growth after cessation of parenteral nutrition (PN) by oral and/or tube feeding for 3 months (10).

Possible influences of intestinal adaption in pediatric IF patients include diagnostic and anatomical factors, such as necrotizing enterocolitis (NEC), residual small intestine, residual colon length, and ileocecal valve (ICV) dysfunction, as well as biochemical factors, such as plasma citrulline (11–13), timing of colostomy closure, timing and progress of enteral feeding, imbalance in intestinal bacteria, and the incidence of complications, such as IF-associated liver disease (IFALD) and catheter-related bloodstream infections (CRBSIs) (10–15), which can adversely affect intestinal adaption and increase the risk of mortality (16), although sufficient evidence is currently lacking.

The aims of the present study were to identify predictors of survival and enteral autonomy, as well as risk factors of IF-related complications by a multi-center pediatric cohort in China.

## Patients and Methods

### Study Approval

The study protocol was approved by Ethics Committees of all participating hospitals and conducted in accordance with the tenets of the Declaration of Helsinki. Informed consent was obtained from the parents/guardians of the children after receiving an explanation of the purpose if the study.

### Study Population

The cohort of this retrospective study included 92 children with IF who received treatment at the Children's Hospital of Nanjing Medical University (Nanjing, China), Xinhua Hospital Affiliated to Shanghai Jiao Tong University School of Medicine (Shanghai, China), Guangzhou Women and Children's Medical Center (Guangzhou, China), and Hunan Children's Hospital (Changsha, China) from January 1, 2012 to November 31, 2020.

All patients included in this study met the following inclusion criteria: severe congenital or acquired gastrointestinal diseases; age, <4 years; and PN duration of >42 consecutive days. Enteral autonomy was defined as maintaining sufficient growth after cessation of all PN by oral and/or tube feeding for 3 consecutive months. The exclusion criteria were incomplete clinical data and loss to follow-up after discharge.

### Methods

Variables recorded were general information (sex, birth weight, age, family status, occupation of parents, and family history of related diseases), disease-related information (cause of IF, presence of ICV, age at time of surgery, surgical method, percentages of remaining small bowel, and restoration of bowel continuity), enteral nutrition information (enteral nutrition method and formula), parenteral nutrition information (duration of parenteral nutrition time, selection of fat emulsion in parenteral nutrition, and mode of intravenous nutrition), and complications and laboratory indicators during treatment (presence of septicemia or sepsis, maximum leukocyte count, highest level of procalcitonin, presence of IF-related liver disease, and presence of renal failure). Enteral autonomy was defined as sustained growth and cessation of parenteral nutrition for ≥ 90 days. Regular follow-ups were conducted in person at the Outpatient Department or by telephone to February 28, 2021. The primary outcomes of this study were enteral autonomy, persistent dependency on PN, and death. The median follow-up time was 5.2years.

All clinical data were collected and sorted by specially trained personnel assigned by each hospital, and then sent to the Children's Hospital Affiliated to Nanjing Medical University. The personnel were responsible for carefully reviewing and entering the data into the database. For data in question, the project leader of each unit determined whether the data were correct and suitable for interpretation and analysis.

### Statistical Analysis

All statistical analyses were conducted using IBM SPSS Statistics for Windows, version 24.0. (IBM Corporation, Armonk, NY USA). Continuous non-parametric data are presented as the median and inter quartile range (IQR). The Kruskal–Wallis H test was applied for comparisons of three groups, while the two-tailed Mann–Whitney U test was used for comparisons of two groups. Categorical data were compared using the chi-square test and are summarized as the frequency and percentage. A multivariate logistic regression model was used to identify risk factors for enteral autonomy. A probability (*p*) value of < 0.05 was considered statistically significant.

## Results

### Study Population

**Figure 1:**
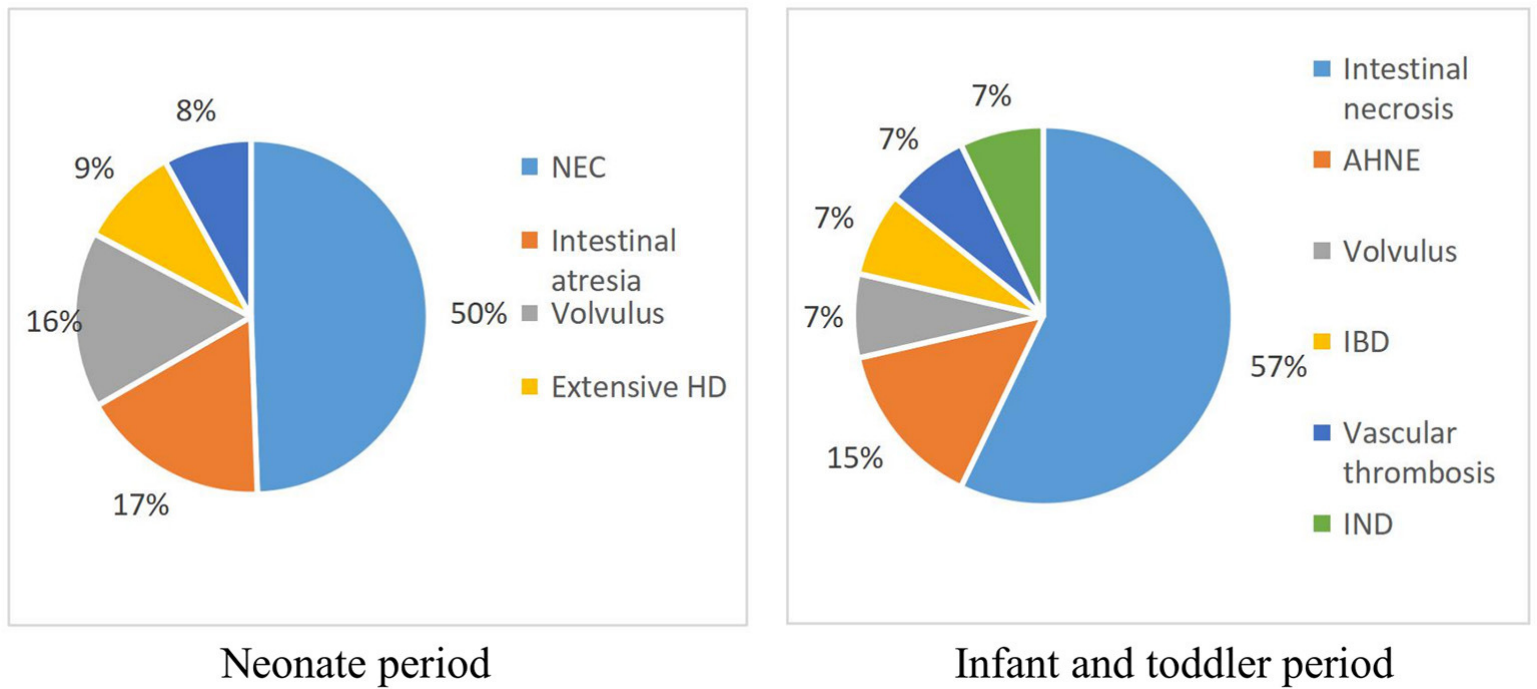
Proportions of the different diagnosis in the study group. AHNE, acute hemorrhagic necrotic enteritis; HD, Hirschsprung disease; IBD, inflammatory bowel disease; IND, intestinal neuronal dysplasia;NEC, necrotizing enterocolitis.

The baseline information of the 92 pediatric IF patients is shown in Table 1. The majority (65.2%) of patients were male and more than half (58.7%) were preterm. The median birth weight was 2235.0 grams (IQR = 1.3500, 3.0875). Most cases (84.8%) occurred during the neonatal period. NEC was the most common cause of IF (*n* = 43), followed by intestinal atresia (*n* = 14), volvulus (*n* = 14), intestinal necrosis (*n* = 8), and extensive Hirschsprung disease (*n* = 7). Details of the causes of IF in infants and toddlers are shown in Figure 1.

**Table 1:** Descriptive statistics of the study population.

**Variable**	**[*N* (%) or median (IQR)]**
No. of patients	92
Female sex	32 (34.8%)
Preterm	54 (58.7%)
Birth weight, grams	2235.0 (1350.0, 3087.5)
Age of onset, days	8.00 (1.00, 33.75)
Disease occurred in the neonatal period	78 (84.8%)
**Operation information**	
No. of patients undergoing surgery	71 (77.2%)
No. of laparotomies	1 (1,2)
RSB, cm (*n* = 57)	80 (60, 100)
Presence of ICV (*n* = 57)	31 (54.4%)
**EN information**	
Human milk	38 (41.3%)
Type of formula milk	
Whole protein formulas	13
pHF	10
eHF	67
AAF	1
EN route	
Oral feeding	52 (56.5%)
Nasal feeding	40 (43.5%)
**PN information**	
Lipid emulsion	
MCT/LCT	45 (48.9%)
SMOF	47 (51.1%)
Venous access	
CVC	11 (12.0%)
PICC	42 (45.7%)
PVC	39 (42.4%)
Duration of PN time (months)	2.0 (1.6–5.0)
Major complications	
Sepsis	37 (40.2%)
IFALD	14 (15.2%)
CRBSIs	5 (5.4%)
Patients alive	79 (85.9%)
Enteral autonomy	63 (68.5%)
Median follow-up time (years)	5.2

*AAF, Amino acid-based formulas; CRBSIs, Catheter-related bloodstream infections; CVC, Central vein catheter; eHF, Extensively hydrolyzed protein formula; EN, Enteral nutrition; ICV, Ileocecal valve; IFALD, Intestinal Failure-Associated Liver Disease; MCT/LCT, Medium-chain triacylglycerol / long-chain triacylglycerol; pHF, Partially hydrolyzed protein formula; PICC, Peripherally inserted central catheter; PN, Parenteral nutrition; PVC, Peripheral vein catheter; RSB, Residual small bowel length; SMOF, Olive oil-based lipid emulsion formulas of soybean oil, medium-chain triglycerides, olive oil, and fish oil*.

By the end of the follow-up period (February 28, 2021), 63 (68.5%) patients finally achieved enteral autonomy and 13 (14.1%) died. The causes of death included sepsis, severe pneumonia, Takayasu arteritis, chronic hepatic failure, septic shock, multiple organ dysfunction syndrome, and other unknown factors (Some parents gave up treatment and took children home directly. These children eventually died of some unclear reason).

### Different Treatment Modalities and Analysis of Outcomes

Of the 92 patients, 71 (77.2%) underwent surgery and 21 (22.8%) received non-surgical treatment (Table 2). The surgical patients underwent at least one laparotomy procedure. The surgical procedures included intestinal resection, intestinal anastomosis, enterostomy, lysis of intestinal adhesions, intestinal repair, intestinal biopsy, and abdominal irrigation and drainage. Of the 71 surgery patients, 45 (63.4%) underwent enterostomy as the initial surgery and 26 (36.6%) received entero-anastomosis treatment. Patients in the non-surgical treatment group had a lower mean birth weight, an earlier age of onset, a higher proportion of premature births, a higher proportion of NEC, a shorter PN duration, and a higher proportion of enteral autonomy, whereas patients in the enterostomy group had a higher rate of breast-feeding.

**Table 2:** Clinical statistics of the study population in different subgroups of treatment with pediatric intestinal failure.

**Variable**	**Non-surgical group**	**Enterostomy group**	**Entero-anastomosis group**	***P*-value**
	**(*n* = 21)**	**(*n* = 45)**	**(*n* = 26)**	
	**[*****N*** **or median (IQR)]**			
Female sex	6	16	10	0.811
Birth weight, grams	1292.5 (1090.0, 1617.5)	2700.0 (1650.0, 3150.0)	2.34 (1.58, 3.1)	0.001
Preterm	18	21	15	0.011
Age of onset, days	1.5 (0, 26)	10 (2,34)	9 (1,33)	0.008
Disease occurred in the neonatal period	19	37	22	0.699
Diagnosis				0.002
Necrotizing enterocolitis	18	19	6	
Intestinal atresia	0	6	8	
Volvulus	1	7	6	
Extensive HD	0	4	3	
Intestinal necrosis	0	6	2	
Other diagnosis	2	3	1	
**Operation information**				
RSB, cm	-	80 (60, 100)	80 (60, 100)	0.050
Presence of ICV	-	19 (57.6%)	12 (50%)	0.571
Initial operation age, days	-	8.5 (4,30)	8 (4,30)	0.729
Human milk	5	25	8	0.022
SMOF	14	21	12	0.293
Duration of PN time, months	1.55 (1.4, 1.925)	3 (2,5)	3 (1.8, 5)	0.000
Mortality	3	8	2	0.537
Major-cause complication				
Sepsis	12	17	8	0.158
IFALD	4	8	2	0.485
CRBSIs	1	3	1	1.000

*CRBSIs, Catheter-related bloodstream infections; HD, Hirschsprung disease; ICV, Ileocecal valve;IFALD, Intestinal Failure-Associated Liver Disease; PN, Parenteral nutrition; RSB, Residual small bowel length; SMOF, Olive oil-based lipid emulsion formulas of soybean oil, medium-chain triglycerides, olive oil, and fish oil*.

Specifically, 46 patients underwent only one surgery throughout the whole treatment period, which included 27 who achieved enteral autonomy, while 17 underwent two surgeries (13 cases finally achieved enteral autonomy), which mainly included lysis of intestinal adhesions, intestinal resection, and enterostomy. In addition, five patients underwent three surgeries (two cases finally achieved enteral autonomy) due to intestinal adhesions, intestinal stenosis, and ileum duplication. Three children underwent four surgeries across the duration of the hospital stay.

### Enteral Autonomy

By the end of the follow-up period, 63 (68.5%) patients had achieved complete enteral autonomy, 16 (17.4%) were still partially or completely dependent on PN, and 13 (14.1%) had died.

As shown in Table 3, clinical factors associated with enteral autonomy included a relatively short period of PN, feeding of human milk, and use of SMOF-lipids (Fresenius Kabi, Austria). Conversely, sepsis was associated with a lower likelihood of achieving enteral autonomy, while the presence of ICV and greater residual small bowel (RSB) length were significantly associated with enteral autonomy. In addition, 33 children with an underlying diagnosis of NEC eventually achieved enteral autonomy, yet no significant difference was found between NEC group and other disease groups.

**Table 3:** Baseline characteristics based on the primary outcome of achievement of enteral autonomy in children with intestinal failure.

**Variable**	**Achieved enteral autonomy** **(*n* = 63)**	**Did not achieve enteral autonomy** **(*n* = 29)**	***P*-value**
	**[N or median (IQR)]**		
Female sex	21	11	0.667
Birth weight, grams	2260.0 (1352.5, 3087.5)	2235.0 (1350.0, 3062.5)	0.246
Preterm	38	16	0.641
Age of onset, days	8 (1, 32.75)	8 (1, 33.25)	0.141
Disease occurred in the neonatal period	56	22	0.192
Diagnosis			0.110
Necrotizing enterocolitis	33	10	
Other diagnosis	30	19	
RSB, cm	80 (60, 100)	80 (60, 100)	0.000
Presence of ICV	22 (68.75%)	9 (36%)	0.014
Initial operation age, days	8.5 (4,30)	9 (4,30)	0.586
No. of laparotomies	1 (1,2)	1 (1,2)	0.388
Human milk	33	5	0.001
Duration of PN time, months	2.1 (1.6, 5)	2.1 (1.6, 5)	0.000
Lipid emulsion			0.031
MCT/LCT	26	19	
SMOF	37	10	
Venous access			0.449
PVC	26	13	
CVC	6	5	
PICC	31	11	
Major-cause complication			
Sepsis	20	17	0.015
IFALD	7	7	0.192
CRBSIs	2	3	0.321

*CRBSIs, Catheter-related bloodstream infections; CVC, Central vein catheter; IFALD, Intestinal Failure-Associated Liver Disease; ICV, Ileocecal valve; MCT/LCT, Medium-chain triacylglycerol / long-chain triacylglycerol; PN, Parenteral nutrition; PICC, Peripherally inserted central catheter; PVC, Peripheral vein catheter; RSB, Residual small bowel length; SMOF, Olive oil-based lipid emulsion formulas of soybean oil, medium-chain triglycerides, olive oil, and fish oil*.

Multivariate logistic regression analysis of the whole cohort (*n* = 92) revealed that longer PN duration (OR = 0.75, 95% CI = 0.565–0.997), and sepsis (OR = 0.277, 95% CI = 0.082–0.938) were risk factors for enteral autonomy, while breastfeeding (OR = 5.295, 95% CI = 1.445–19.404) was a protective factor (Table 4).

**Table 4:** Multivariable logistic regression analysis based on the primary outcome of achievement of enteral autonomy in children with intestinal failure.

**Model**	**Characteristics**	**B value**	***OR* value [Exp (B)]**	**95%*CI***	***P-*value**
Model 1,	**Duration of PN time**	−0.287	0.750	0.565–0.997	0.047
all children, *n* = 92	**Sepsis**				
	NO	Reference	-	-	-
	YES	−1.285	0.277	0.082–0.938	0.039
	**EN composition**				
	Formula milk	Reference	-	-	-
	Human milk	1.667	5.295	1.445–19.404	0.012
Model 2,	**RSB**	0.050	1.051	1.008–1.097	0.021
measured small bowel	**ICV**				
length and ICV,	NO	Reference	-	-	-
*n* = 57	YES	2.096	8.131	1.321–50.038	0.024

*Model 1 includes data from all 92 children; model 2 includes only those children with intra-operatively measured residual small bowel length (n = 57). CI, Confidence interval; EN, Enteral nutrition; ICV, Ileocecal valve; OR, Odds Ratio; PN, Parenteral nutrition; RSB, Residual small bowel length*.

Further multivariate logistic regression analysis of 57 children with data regarding RSB length and the presence or absence of an ileocecal valve showed that RSB length (OR = 1.051,95% CI = 1.008–1.097) and the presence of an ICV (OR = 8.131,95% CI = 1.321–50.038) were identified as independent factors affecting enteral autonomy. The probability of achieving enteral autonomy increased by 5.1% for each additional 1 cm in RSB length and the presence of ICV was associated with an 8.131-fold greater likelihood of achieving enteral autonomy.

### Risk Factors for Development of IFALD

IFALD was defined as cholestasis occurring in the setting of PN for more than 2 weeks upon exclusion of other specific causes of liver disease (18), while cholestasis was defined as an elevated conjugated serum bilirubin level of > 34.2 mmol/L (19). During the study period, 38 (41.3%) patients were complicated with cholestasis and 14 (15.2%) eventually developed IFALD (Table 5). There were significant differences in lipid emulsion and sepsis between the two groups, and the incidence of IFALD was higher in children treated with medium/long-chain (MCT/LCT) lipids or sepsis. MCT/LCT lipids and sepsis were identified as negative predictors of IFALD. Notably, there was no case of renal failure in this cohort.

**Table 5:** Single factors analysis of IFALD in children with Intestinal Failure.

**Variable**	**IFALD** **(*n* = 14)**	**Non-IFALD** **(*n* = 78)**	***P*-value**
	**[N or median (IQR)]**		
Female sex	6	26	0.701
Preterm	9	45	0.645
Birth weight, grams	2290.0 (1452.5, 3100.0)	2240.0 (1350.0, 3075.0)	0.7
Diagnosis	Not display	Not display	0.194
Age of onset, days	9.5 (1, 35.5)	8 (1, 32.5)	0.268
RSB, cm	80 (60, 100)	80 (60, 100)	0.653
ICV	5 (55.6%)	26 (54.2%)	1.000
Human milk	6	32	0.898
SMOF	1	46	0.000
Duration of PN time, months	2.2 (1.6, 5)	2 (1.6, 5)	0.81
Sepsis	9	28	0.046
CRBSIs	2	3	0.165

*CRBSIs, Catheter-related bloodstream infections; ICV, Ileocecal valve; IFALD, Intestinal Failure-Associated Liver Disease; IQR, Interquartile range; PN, Parenteral nutrition; RSB, Residual small bowel length; SMOF, Olive oil-based lipid emulsion formulas of soybean oil, medium-chain triglycerides, olive oil, and fish oil*.

### Risk Factors for Developing CRBSIs

A central venous catheter (CVC) or peripherally inserted central catheter (PICC) is most commonly used for central venous administration in clinical practice, as stable intravenous infusion channels can guarantee long-term intravenous fluid treatment and greatly alleviate pain due to repeated venipuncture. In this study, a CVC was used for infusion in 11 children, while a PICC was used in 42, and the remaining 39 patients received peripheral intravenous infusion (Table 1). Due to prolonged parenteral nutrition, blood stream infections related to the presence of a CVC are among the most common complications in this population. In total, 37 children had concurrent sepsis, which included 21 with positive blood cultures.

CRBSI was defined as a positive blood culture (one sample from the central venous and a second from a peripheral vein) in combination with clinical infectious symptoms (20). Cultures of blood samples collected from five children with CRBSI revealed the presence of *Klebsiella pneumoniae* (2 cases), *Klebsiella oxytoca, Candida guilliermondii*, and *Candida albicans*. Univariate analysis revealed that the use of MCT/LCT lipids was associated with an increased risk of CRBSI (Table 6). More particularly, although a PICC was used for all 5 patients, there was no significant difference of the incidence of CRBSI between the two groups in regard to venous access.

**Table 6:** Single factors analysis of CRBSIs in children with intestinal failure.

**Variable**	**CRBSIs** **(*n* = 5)**	**Non-CRBSIs** **(*n* = 48)**	***P*-value**
	**[N or median (IQR)]**		
Female sex	2	17	1.000
Preterm	3	32	1.000
Birth weight, grams	2740.0 (1320.0, 3430.0)	1670.0 (1200.0, 3000.0)	0.808
Diagnosis	Not display	Not display	0.136
Age of onset, days	6 (1,29)	7 (0, 34)	0.829
RSB, cm	100 (75, 102.5)	81.25 (57.75, 103.75)	0.831
ICV	1 (25%)	16 (61.5%)	0.290
Human milk	1	18	0.643
SMOF	0	28	0.019
Duration of PN time, months	2 (1.55, 4.75)	2 (1.5, 5)	0.264
Venous access			0.571
CVC	0	11	
PICC	5	37	
Sepsis	5	25	0.061
IFALD	2	7	0.196

*CRBSIs, Catheter-related bloodstream infections; ICV, Ileocecal valve; IFALD, Intestinal Failure-Associated Liver Disease; IQR, Interquartile range; PN, Parenteral nutrition; RSB, Residual small bowel length; SMOF, Olive oil-based lipid emulsion formulas of soybean oil, medium-chain triglycerides, olive oil, and fish oil*.

## Discussion

The main purpose of managing IF in childhood is to increase intestinal adaptation and achieve enteral autonomy, which is defined as complete cessation of PN for more than 90 days and maintaining acceptable growth parameters. The identification of factors associated with the ability to achieve enteral autonomy is very important for the family and medical team. It is also very important to know which factors may lead to IF-related complications to facilitate prevention and treatment.

The duration of intestinal adaptation varies among patients and the most significant progression usually occurs in the first few years after intestinal surgery. In this study, enteral autonomy was achieved in 63 patients (68.5%) at a follow up of ≥ 1 year, which was slightly higher than in previous reports of 43 to 67% (7, 11, 12, 21, 22). This difference may be explained by the high proportion of non-surgical patients (n = 21, 22.8%) in this study, which included 18 (85.7%) who eventually achieved enteral autonomy. Premature neonates, which comprised the majority of the non-surgical group, are known to have strong compensatory abilities and more likely to obtain intestinal adaptation (23). Additionally, since the integrity of the gut of non-surgical patients is not disrupted, intestinal absorption capacity is less affected, thus enteral nutrition can be carried out as early as possible to reduce dependence on parenteral nutrition and achieve enteral autonomy as soon as possible. Although most children were weaned from PN within the first year, intestinal adaptation may continue throughout childhood.

The mechanism underlying intestinal adaptation after intestinal resection remains unclear. However, some studies have found no difference in the probability of weaning infants with NEC from PN (24), while others suggest that infants with NEC are more likely to achieve enteral autonomy than those without (11). Of the 43 children with NEC enrolled in this study, 33 (76.7%) achieved enteral autonomy, suggesting that children with NEC are more likely to achieve enteral autonomy than those without. However, the difference between the two groups was not significant.

Extensive data have established that the length of the residual small intestine and remaining ileocecal valve have significant effects on enteral autonomy (11, 25, 26). Consistent with the findings of previous studies, the results of the present study showed that a longer RSB or the presence of an ileocecal valve was associated with a greater likelihood of achieving enteral autonomy. For every 1 cm increase in RSB length, the probability of achieving enteral autonomy is increased by 5.1%.

Choosing human milk or deep hydrolyzed formula as a source of enteral nutrition may also play a role in intestinal adaptation. Breast milk has many advantages, including secretory IgA, amino acids, and a variety of growth factors that can enhance the systemic and mucosal immune responses, and many guidelines recommend the use of breast milk. Therefore, in the absence of specific intolerance, breast milk should be strongly considered as the main enteral nutrition source. In the present cohort, almost half (41.3%) of the children were breastfeeding, which can promote enteral autonomy. When unavailable, human breast milk can be partly or fully replaced with special hydrolyzed formula (27). In the present study, 67 patients were fed with deep hydrolyzed formula. However, there was no obvious benefit to recovery.

IFALD is an important factor affecting enteral autonomy in children and may progress to end-stage liver disease, which is negatively correlated with survival (28, 29). Two recent large studies of 279 (30) and 251 (17) pediatric IF patients reported that the prevalence of IFALD was 22 and 20%, respectively. However, the prevalence of IFALD (15%) in the present study was less than that of these previous studies. A possible explanation for this discrepancy may be the high proportion of SMOF-lipid use (51.1%). Importantly, the development of IFALD has a notable effect on clinical outcomes of IF patients (31). In this study, IFALD was not correlated with enteral autonomy, although mortality was higher in children with IFALD than without (χ^2^ = 6.34, *p* = 0.0118, detailed data are not included in the table).

The identification of risk factors for IFALD is helpful for the prevention and treatment of IFALD and could benefit prognosis. The results of the present study revealed that the risk factors leading to IFALD included sepsis and intravenous administration of lipids. More specifically, the use of MCT/LCT lipids (IFALD vs. non-IFALD: 13 vs. 32 patients) was associated with a greater risk of IFALD than the use of SMOF-lipids (IFALD vs. non-IFALD: 1 vs. 46 patients) and patients with sepsis were more likely to develop IFALD than those without (IFALD vs. non-IFALD: 64 vs. 36%). Previous studies have reported that the bile ducts and hepatocytes of premature infants are more vulnerable to severe damage (32, 33) and the occurrence of IFALD may be associated with premature birth. In the present study, the proportion of premature infants (59%) was not very high and preterm infants with IF (IFALD vs. non-IFALD: 64 vs. 49%, *p* = 0.645) were not more susceptible to IFALD.

The exact mechanism underlying PN-induced cholestasis remains unclear. In intestinal stasis or intestinal dysfunction, bacteria are transported through the epithelial barrier and release endotoxins (34), consistent with the finding that sepsis is another factor leading to of aggravating IFALD. Endotoxins bind to CD14 receptors on liver macrophages and induce the release of inflammatory cytokines, such as interleukin-1, interleukin-6, and tumor necrosis factor, leading to liver injury (35). When food passes through the gastrointestinal tract, it can increase the expression of insulin-like growth factor and other growth factors, which may improve liver function (36, 37). The lack of enteral food stimulation may reduce cholecystokinin secretion, decrease gallbladder emptying and bile flow, and increase the risk of cholestasis. Fat emulsion, as an important source of energy, plays an important role in the survival and rehabilitation of children. Recent studies have shown that the medium and long chain fat emulsion derived from soybean fat has pro-inflammatory effects, which have been associated with the occurrence of liver diseases related to IF. Long-term conventional infusion of this kind of fat emulsion can easily lead to cholestasis and cirrhosis (38). The new generation of fish oil fat emulsions, such as SMOF-lipids, are rich in omega-3- polyunsaturated fatty acids, which can help to regulate immunity and anti-inflammatory responses, reduce cholestasis and liver damage, and effectively improve the prognosis of children with IF (39).

A CVC is widely used to obtain intravenous access in pediatric patients with IF requiring long-term parenteral nutrition. However, CVC use has been associated with major complications, such as infection, mechanic, and thrombotic consequences (40). In the present study, the median duration of parenteral nutrition was 2 months and both PICCs (45.7%) and CVCs (12.0%) were widely applied. With the increasingly standardized management of venous catheter use, the incidence of CRBSIs has been very low (5% in our cohort). In this study, lipid emulsion was identified as a risk factor for CRBSI. In addition, the administration of MCT/LCT-lipids was associated with an increased risk of CRBSI. As mentioned before, the anti-inflammatory effect of SMOF-lipids is important for children with IF because this population is especially susceptible to infections caused by intestinal flora disorders, which is potentially beneficial to alleviate the inflammatory response and immunosuppression. In general, the major pathogens isolated in patients with CRBSIs are Gram-positive organisms, particularly coagulase-negative *Staphylococcus*, Gram-negative *Enterobacter sp*., *Escherichia coli*, and *Klebsiella sp*., and *fungi* (41, 42). In this study, the pathogens cultured from patients with CRBSIs were similar to those in previous articles. Antibiotic lock therapy has been used to avoid direct removal and rescue the central catheter after CRBSIs. The use of ethanol and standardized nursing care is reported to significantly reduce the incidence of CRBSIs by up to 15% (17, 43, 44).

There were some limitations to this study that should be addressed. First, this was a retrospective study with a relatively small number of patients. Due to the large time span of the study, some important potential factors, such as medication use, remaining colon, etc., could not be completely collected to determine which should be further refined in future studies.

In conclusion, even though patient survival has significantly improved, IF still poses a significant threat to human health. The use of a geographically diverse cohort revealed that a longer PN time and sepsis were risk factors for enteral autonomy, while breastfeeding was a protective factor. When considering the detailed intraoperative data, the presence of ICV and a greater RSB length were reaffirmed as predictors for reaching enteral autonomy. At present, the best strategies to reduce the incidence of IFALD include meticulous care to prevent sepsis and the use of SMOF-lipids. Despite the low incidence of CRBSI, evidence suggests that the use of SMOF-lipids can effectively ameliorate CRBSI. These data are important references for future prospective studies to further improve the outcomes of children with IF.

## Data Availability Statement

The original contributions presented in the study are included in the article/supplementary material, further inquiries can be directed to the corresponding author/s.

## Ethics Statement

The studies involving human participants were reviewed and approved by IEC of Children's Hospital of Nanjing Medical University. Written informed consent to participate in this study was provided by the participants' legal guardian/next of kin.

## Author Contributions

All authors listed have made a substantial, direct, and intellectual contribution to the work and approved it for publication.

## Funding

This study was funded by Key Project of Science and Technology Development of Nanjing health committee, China (ZKX18037); Jiangsu Youth Medical Talent Project, China (QNRC2016081);Top Talents of Jiangsu Provincial Health Committee's Six One Project, China (LGY2020019); the Jiangsu Provincial Key Research and Development Program, China (BE2017609) and General project of Nanjing Health Commission (YKK20121).

## Conflict of Interest

The authors declare that the research was conducted in the absence of any commercial or financial relationships that could be construed as a potential conflict of interest.

## Publisher's Note

All claims expressed in this article are solely those of the authors and do not necessarily represent those of their affiliated organizations, or those of the publisher, the editors and the reviewers. Any product that may be evaluated in this article, or claim that may be made by its manufacturer, is not guaranteed or endorsed by the publisher.

## Abbreviations

IF: Intestinal failure
ICV: Ileocecal valve
EN: Enteral nutrition
PN: Parenteral nutrition
SMOF: Olive oil-based lipid emulsion formulas of soybean oil, medium-chain triglycerides, olive oil, and fish oil
MCT/LCT: Medium-chain triacylglycerol / long-chain triacylglycerol
PVC: Peripheral vein catheter
CVC: Central vein catheter
PICC: Peripherally inserted central catheter
HD: Hirschsprung disease
RSB: Residual small bowel length
OR: Odds Ratio
CI: Confidence interval
CRBSIs: Catheter-related bloodstream infections
IFALD: Intestinal Failure-Associated Liver Disease.
